# Sequences and Structures of Viral Proteins Linked to the Genomes (VPg) of RNA Viruses

**DOI:** 10.3390/v17050645

**Published:** 2025-04-29

**Authors:** Catherine H. Schein

**Affiliations:** Department of Biochemistry and Molecular Biology, University of Texas Medical Branch, Galveston, TX 77555, USA; chschein@utmb.edu

**Keywords:** Picornaviridae, enterovirus, kobuvirus, dicistrovirus, comoviruses, calicivirus, norovirus, potyvirus, VPg sequences and structures, motifs and hidden Markov models (HMM), conserved lysines, N-degrons

## Abstract

In the mid-1970s, it was revealed that the 5′ end of the RNA genome of poliovirus (PV) was covalently linked to a peptide called VPg (viral protein, genome-linked). Subsequently, VPgs have been found attached to many other viruses and even phages. This review summarizes the patterns of physicochemical properties that are conserved within the VPgs of plus-strand RNA viruses where short-peptide VPgs have been identified. Mutagenesis and structural data indicate the importance of a 5 aa conserved motif at the N-termini of picornaviral VPgs (around the tyrosine 3 residue, which forms a covalent bond to UMP and the RNA). Hidden Markov models have been used to find motifs and VPgs in additional genera of picornaviruses, as well as dicistroviruses in insects and comoviruses in plants. These latter VPgs are bound to the RNA termina through linkages to serine or threonine. The role of free VPg and VPgpU needs clarification, especially in light of multiple genome copies in many of the viruses. Lysine and other positively charged side chains are hallmarks of VPgs. These may contribute to interactions with the viral RNA, polymerase, membranes and cellular proteins. The larger protein VPgs from potyviruses and noroviruses/caliciviruses may also show some areas of similar properties to these small peptides.

## 1. Introduction

Early researchers found that the RNA genome of poliovirus (PV) was covalently bound to a small, 22-amino-acid-long peptide at its 5′ end [1,2], called the “protein linked to the genome” or VPg. VPgs were soon demonstrated for other plant and animal viruses [3]. VPgs replace the cap structure of cellular RNA, priming for viral genome replication while also limiting the immune response to nucleotides with a free 5′ end [4]. While larger VPgs were also identified in other virus groups, such as potyviruses [5] and caliciviruses [6], including noroviruses, the majority of research has been performed on the short-peptide VPgs of enteroviruses (especially poliovirus (PV)) and the aphthovirus foot and mouth disease (FMDV). This is largely because enteroviruses (of which there are now over 10,000 sequences in databases) were one of the first virus groups to be isolated and studied. They include, besides PV, viruses referred to as coxsackie A or B (named for Coxsackie, New York, where they were first isolated [7]), rhinoviruses and many other human and animal pathogens. Agriculture can be greatly affected by outbreaks of dicistroviruses (affecting bees and shrimp [8], although they have also been found associated with domestic and wild animals, including squirrels and raccoon dogs [9]) and comoviruses [10], which infect beans, potatoes and many other crops. This minireview compares the sequences of VPg peptides and distantly related ones from dicistroviruses and some plant viruses. The conserved features of the small VPgs (up to about 30 amino acids) reveal motifs involved in nucleotidylation and may also suggest how these peptides interact with the viral RNA, polymerases, cell membranes and proteins. There are experimental structures of VPg peptides from poliovirus and partial structures from other enteroviruses, which are lacking for the other viruses.

### 1.1. Picornavirus VPgs Are Essential for Transcription

**Figure 1:**
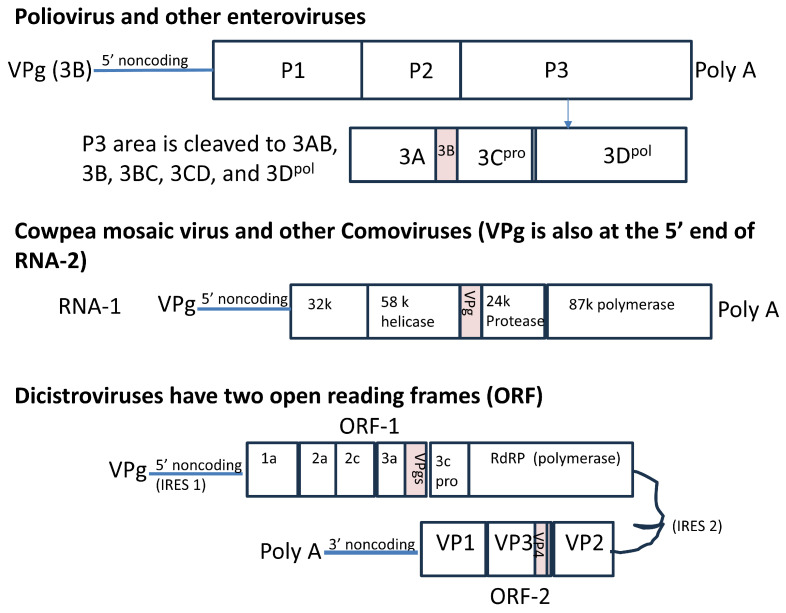
VPgs, attached to the 5′ end of the genome, are encoded within the viral genome. The position of the VPg (protein 3B) next to the 3C protease and 3D polymerase genes in the poliovirus genome is shown; the exact positions of the *cre* and IRES differ in the picornaviruses. The VPg genes are found in RNA-1 of cowpea mosaic virus [14], although both RNAs are linked to VPg, and in ORF-1 of cricket paralysis virus [15]. Dicistroviruses can contain up to 8 copies of VPgs. The longer coding sequences of the VPgs of potyviruses [16] and caliciviruses [17] are also found to precede the viral polymerase-coding region. The drawing is not to scale. The folding of the RNA 5′ and 3′ non-coding regions/poly A regions, which play a role in VPg binding [18], is also not indicated.

The RNA of poliovirus (PV) and other picornaviruses is first translated as one protein, which is subsequently cleaved by cis-encoded proteases into three proteins, P1–P3. Although found at the start of the RNA, the “viral protein linked to the genome”, VPg, or 3B, is cleaved from the middle of the last section of the polyprotein, called P3 (Figure 1). The VPgs of picornaviruses are uridylylated, and the 5′ nucleotide of their genomic RNA is always U. Uridylylation to VPgpUpU (and longer stretches of poly U) can be catalyzed in a reaction containing only the viral polymerase, poly A, Mn^++^, UTP and the VPg peptide. The reaction can be made much more efficient if a small hairpin region from within the RNA, called the cis-acting replication element (*cre*), is added to the reaction as the template RNA (rather than poly A). Although the *cre* in PV is located within the coding sequence for 2C, it is found within the 2A region for rhinoviruses, and its position varies greatly in other picornaviruses. Its position in the aphthovirus foot and mouth disease virus (FMDV) is in the 5′ non-coding region, near the internal ribosome entry site (IRES) [11]. While mutating the *cre* greatly reduces replication, the element can be moved to the end of the FMDV genome without affecting replication. While the genome locations of *cre* and IRES elements, as well as their interactions with host proteins [12], are virus-dependent [13], the genes for VPgs are consistently located near the polymerase genes, even in quite different viruses.

The VPgs identified for the over 200 serotypes of enteroviruses show remarkable conservation in length and charge. They have been further divided into A–J groups, consisting of viruses such as polio, coxsackie and echoviruses [19], and three groups of rhinoviruses (originally recognized as a discrete genus of picornaviruses, these are now within the enterovirus genus based on their similar genome organization [20,21]). The Rhinoviruses were originally thought to only cause respiratory ailments such as the common cold, until it was found that EV-D68 (first called HRV-87 [22]) could cause paralysis in children [23]. A similar paralytic syndrome in a murine model of EV-D68 infection has been mapped to residues in the P1 region [23] (see Figure 1). The VPgs of rhinoviruses have a slightly lower net charge at pH 7 due to negatively charged residues (glutamic acid, E) compared to other enteroviruses (which can quickly be seen by comparison to the physicochemical property (PCP) consensus of enteroviruses [24] in Table 1), but a similarly high isoelectric point (IEP). As Table 1 shows, most rhinoviral VPgs share some characteristics with those of the aphthovirus FMDV. However, the VPg of EV-D68 is more similar to those of PV-associated enteroviruses, as it has isoleucine (I) or leucine (L) in place of glutamates, showing, again, that it is indeed not a “typical rhinovirus”, i.e., one that causes largely respiratory ailments.

We now know much about how picornaviral VPg folds [30], is uridylylated [31] to VPgpUpU [32,33] and interacts with the viral RNA and *cre* [34], the viral polymerase and the cellular transcription machinery [35]. VPg primes both coding and template (plus and minus) strand RNA synthesis. While the attachment of VPg is needed for plus-strand transcription, a cellular protein (previously identified as a DNA repair enzyme) may remove the peptide before translation [36,37]. However, viral replication is initially slowed, but not eliminated, if VPg cleavage from the RNA is blocked.

The attachment of VPg is also needed for the transcription of the minus strand of the virus, although the mechanism for this may require a different model of the complex of proteins and RNA than for attachment to the plus strand [34]. Interestingly, VPg uridylylation and negative-strand synthesis are coordinated in time and reliant on a mechanism that is inactivated by low (2 mM) guanidinium concentrations. That is, the two processes start and stop simultaneously upon the addition or removal of the guanidium [38]. These models also account for the need for a long poly A tail (the end of which would base-pair with VPgpUpU and longer, polyuridylylated-VPg) on the RNA for efficient virus replication [39]. This has been noted by many groups attempting to produce an infectious virus from plasmids, including the finding that a length of at least 14 poly A was needed in the DNA to obtain Seneca picornavirus [40]. The authors also note that much longer poly A tracts were found in the isolated viral RNA after 5–10 rounds of replication.

An early model for the uridylylation of VPg on the PV polymerase surface suggested that poly A synthesized from the replication site within the polymerase would need to be of sufficient length (at least nine bases) to be able to serve as a template for the uridylylation reaction [31]. This mechanism for uridylylation using a poly A RNA template, positing a surface of the 3D polymerase for binding, was based on data from the structural analysis and substitution of individual residues of the PV polymerase [31]. The crystal structures of several enterovirus and picornaviral polymerases complexed with their VPgs have indicated that there are indeed surface-binding sites on their polymerases that could play a role in uridylylation. However, due to the flexibility of the peptide, the complete structures of VPgs bound to their cognate polymerases have been difficult to distinguish [41,42,43,44].

### 1.2. Relationship Between Sequence and Structure of Picornaviral VPg

**Figure 2:**
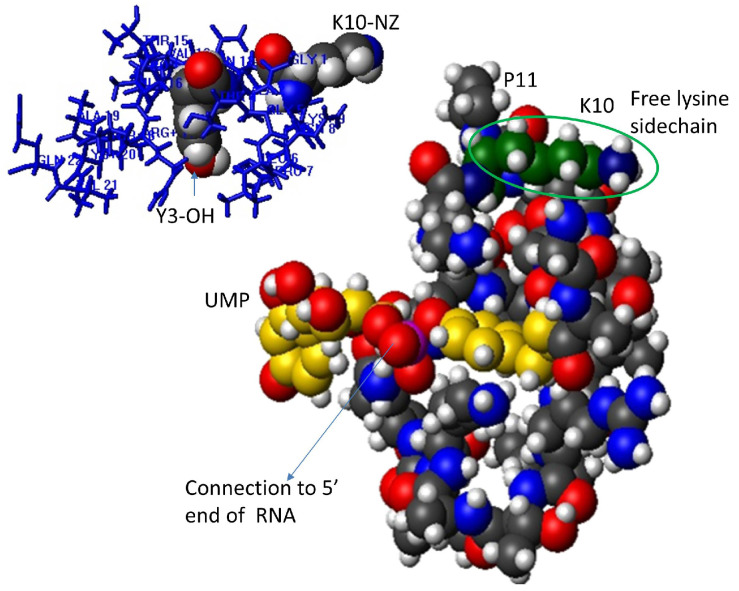
**Top left**: PV-VPg NMR structure (1BBV.001). The residues are shown as blue lines, but Y3 and K10 are space-filling and colored per atom (C atoms are colored black, P magenta, O red and N blue). **Below**: NMR structure bundle for PV-VPgpU [33], with the H atoms shown as small gray spheres; the atom radii of other atoms were set to 1. The C atoms of the uridylylated tyrosine (TYU3) are colored gold; those of K10 (circled) are colored green.

In PV and other picornaviruses, a tyrosine (Y) residue forms a phosphodiester link to the terminal uridine of the coding sequence [45,46]. Relatively large quantities of diuridylylated peptide (VPgpUpU) are also found free in infected cells [47]. The tyrosine at position 3 (Y3), which would be coupled to the 5′-terminal uridine of the RNA, as well as positively charged residues throughout the peptide [48], were shown to be essential for efficient viral transcription. As the top part of Table 1 shows, the absolutely conserved, uridylylated Tyr (TYU) at position 3 is in a five-residue motif **G**(A/P)**Y**(S/T/A)**G,** conserved throughout the enteroviruses and in FMDV. The choice of the second and fourth amino acids is conserved in each species of enterovirus. The motif changes in kobuvirus VPgs (Table 1) but Y is still at position 3. This N-terminal region contains the site of uridylylation/RNA attachment, although even residues at the far C-terminus may fold back to support the uridylylation of the modified Y3 ([33] and Figure 2). The positions of the Y3 and T4 residues cannot be switched in PV-VPg or replaced with phenylalanine (F) in FMDV or PV VPgs [48,49], emphasizing the importance of both the sequence and structure in VPg binding to nucleotides. VPgs of different enteroviruses have similar physicochemical properties (PCPs) [28,50,51,52,53,54] and can even be uridylylated by quite different enteroviral polymerases [24].

### 1.3. VPgs Have Co-Evolved with Their Proteases and Polymerases

In the picornaviruses, mutations that prevent polyprotein cleavage also affect replication. When the cleavage site between PV-VPg and 3C was changed from Gln-Gly to Gly-Gly (the “GG” substitution), the viral RNA became bound to the uncleaved 3BC protein. While the RNA could be replicated and the polyprotein (other than the GG site) processed, the yield of infectious virus was greatly reduced [55]. It has been repeatedly demonstrated that the RNA transcription of the virus depends on efficient coupling to VPg. However, Coxsackie B viruses with 5′ deletions (i.e., removing the binding sites on the RNA for VPgs) can continue to damage infected heart cells [56]. Their replication is limited and, as the authors suggest, dependent on tiny quantities of intact virus (which may infect subsequent heart transplants). However, the shortened RNAs can be translated by the cellular protein complexes recruited to their IRES elements. Thus, the expression of the tissue-damaging viral proteases can continue, as well as other viral proteins that hinder host cell metabolism, even in the absence of bound VPg.

Differences in the specificity for nucleotide binding of the VPgs of picornaviruses can also be related to differences in the first amino acids in the sequences of their polymerase partners [43,44]. Kobuvirus polymerases, e.g., Aichi virus and sicinivirus, are distinct from other picornaviruses in having a different amino acid (S instead of G) at their N-termini that interacts with the metal-binding residues in the middle of the polymerase [57]. These viruses both have VPgs [58] that are intermediate in sequence between the conserved features of the enterovirus VPgs [24] and those of FMDV (see Table 1).

### 1.4. The Short VPgs of Plant Comoviruses and Insect Discistroviruses Have Distinct Sequences and Predicted Structures

As the sequencing of whole viruses [59] became routine [60], many different viruses were found to have VPgs that were covalently bound [3]. However, the sequencing of the protein bound to RNA was more complicated. Most of the VPgs listed in Table 2 were found by genome sequence homology to those identified by protein sequencing (as was conducted for cowpea mosaic virus, CPMV [14]). The short VPgs of plant comoviruses and insect dicistroviruses are compared in Table 2. The similar genome organization (Figure 1) suggests that these viruses may be distantly related to picornaviruses, but their VPgs are quite different.

A major difference is that the VPgs are linked to RNA via a serine or threonine hydroxyl rather than that of a tyrosine. Serine (S) and threonine (T) are more common residues containing a reactive hydroxyl group in their side chains than Y. These are also the most common phosphorylated amino acids in cells [63]. While the linkage to a hydroxyl group may have a similar mechanism to that of the enteroviruses, the larger and more hydrophobic side chain of Y may allow for better recognition by the cognate polymerase and specificity [64,65].

Although their sequences bear little direct identity to the picornaviruses, comoviral VPgs are relatively well conserved within each species and they have a net positive charge. Their N-termini contain a conserved motif S(R/k)KPNR. The VPg of CPMV was partially sequenced after it was cleaved from the end of the viral RNA; the exact coding sequence was then discerned from the viral gene sequence [62].

The dicistrovirus VPgs, on the other hand, show considerable sequence diversity and even length. They lack a consistent motif for binding to the RNA throughout and show diversity from one isolate to another, although, within each virus, the sequences of their multiple VPgs indicate patterns of conserved residues. The N-terminal sequencing of the protein cleaved from Plautia stali (stink bug) intestinal virus (PTIF) indicated that the VPg peptide began with SQXKXG, where the Xs were undetermined amino acids. There were three possible matches in the genomic RNA—SQEKEG, SQEKIG and SQEKLG—and the authors subsequently determined that these correlated with triplet VPg sequences encoded in the RNA genome [61], similarly to the situation in FMDV. However, other dicistroviruses had quite different sequences and different overall properties. Cricket paralysis virus (CrPV), for example, has a completely different N-terminus and has been shown by mass-spectroscopic analysis to add uridine to the (largely conserved) fourth serine (primarily) but also T9 [15].

A conserved feature of all these VPg sequences is multiple lysine residues. The highly conserved VPgs of picornaviruses and comoviruses have very high IEPs and net positive charges at pH 7, even though the amino acid coupling to the RNA is different. These free lysines and the high net charge of the peptides could aid in several functions, as will be discussed below.

### 1.5. Current Experimental Structures for VPgs

The NMR structures of chemically synthesized [66] PV-VPg and PV-VPgpU indicate how the free lysine side chain could be involved in nucleotidylation, attachment to the viral genome and/or ubiquitin coupling [67,68]. One lysine (K9) lies close to the Y3 that will connect to RNA or be uridylylated, while the side chain of K10 [30,31,33] projects in the opposite direction (Figure 2, top). Substituting this K with arginine (R) prevents or slows replication (depending on a second change of R20) but not uridylylation [48,69]. In the top VPgpU NMR structure (Figure 2, bottom), the K10 amide in the side chain is in an exposed position, projecting away from the UMP attached to Y3 (which also marks the attachment site of the viral RNA) [33]. This position would allow easy interaction of proteins, membranes or nucleic acids with K10, even in the uridylylated or RNA-linked peptide.

Complete experimental structures for VPgs from other viruses are lacking.

### 1.6. How Many VPg Genes/Proteins Does One Virus Need?

Enteroviruses sequenced from many different sources have a single copy of VPg in their genomes, consistent with Figure 1. A single copy in the genome is still sufficient for PV-infected cells to contain free VPg peptides with varying degrees of uridylylation in their cytoplasm [47]. Mutations that affect the uridylylation reaction can be separated from those that affect RNA binding and replication [31,70,71]. However, other picornavirus species have several non-identical genes for VPg, following one another in the viral genome. There are three different VPgs in the aphthovirus FMDV’s genome and two each in mosaviruses, isolated first from the feces of mice, and aquamaviruses, isolated from seals (Table 1).

The question of what purpose the additional VPgs serve is especially relevant when it comes to the dicistroviruses (Table 2) [61], which, as noted above, contain multiple copies of genes for VPg. Using a hidden Markov model (HMM) approach, up to eight copies of VPg have been detected in the aligned protein sequences of dicistroviruses [27]. These additional copies may enhance replication, as indicated by studies in FMDV [49,72] and the dicistrovirus CrPV [15]. While three copies of VPg were additive in FMDV, adding a fourth copy was not helpful and may have hindered replication. Further, different specificities were found for each of the three VPgs, with one being responsible for cis replication, while another could be used in trans (i.e., not from the replicating virus genome). Previous studies also indicated that the three VPgs differed in their roles, with the FMDV VPg3 being the most efficiently uridylylated [73].

Clues in their genomes suggest that the ancestor of the rapidly evolving enteroviruses might originally have had more copies, perhaps resembling the duplicated VPgs that are found in other genera of picornaviruses. Both PV and CVB3 have a sequence immediately following VPg into the P3-C region, similar in its amino acid sequence features to functional VPgs (Table 1). Furthermore, a hydrophobic region immediately preceding the VPg in PV-3AB aids in efficient interaction with the 5′ untranslated region (UTR) of the genomic RNA [74]. However, duplicating the VPg of PV was not successful, as the virus deleted the second copy during replication [75].

### 1.7. What Is the Role of Free VPg, VPgpUpU and Polyuridylylated VPg?

This is a very important question, especially as some viruses encode multiple genome copies, suggesting that the overproduction of the peptide has some advantage for the virus beyond serving as a primer for RNA synthesis. The one aspect that all VPg peptides have in common is positively charged residues, even when the overall net charge is not as high as in the enteroviruses. Notably, the dicistroviruses have quite different overall charges and considerably more diversity in sequences from one virus to another. However, as the VPgs of PSIV and CrPV in Table 2 illustrate, the pattern of positively charged residues (lysines and arginines) is conserved in the multiple copies of VPgs within each virus. This suggests that positively charged residues contribute to the function of these small peptides. As Figure 2 illustrates, the lysine 10 side chain projects outward from the nucleotidylation site, where the VPg becomes covalently attached to RNA.

One obvious reason for the need for positively charged residues is interaction with sites on RNA [70] during the establishment of the covalently linked VPg–RNA complex. The positive charges could also aid in the membrane localization of the RNA complex during transcription [76]. Such an interaction could also aid in the transport of the viral RNA and 3D^pol^ to the membrane vesicles for replication [58]. PV requires membrane association in order to replicate efficiently [76] and indeed causes infected cells to fill up with membranous tissue during the height of replication [77]. PV replication and intracellular transport is inhibited by Brefeldin A, a potent inhibitor of autophagy [78] and cell transport [79].

Another possible reason for the persistence of lysine side chains is that VPgs could be ideal N-degrons. They could hinder autophagy by luring ubiquitinating enzymes, which couple ubiquitin chains to free lysine side chains [80,81,82] to mark the bound protein for proteolysis. Like autophagosomes [83], replicative vesicles for picornaviruses emerge from the ER and Golgi of infected cells. However, while enteroviruses “hide” within membrane complexes [76], taking over nucleic acid metabolism [84] and the translation of proteins, they limit autophagy by degrading the ULK1 protein that initiates it [77,85], using an alternative pathway for autophagy [67,77]. PV-VPg should resist N-terminal proteolysis, as its first amino acids are protease-resistant glycine (G1) alanine at the second position (A2).

### 1.8. Larger VPgs Have Quite Different Sequences and an Expanded Role in Viral Replication

Other VPgs, bound to the genome and essential for replication, were found that varied greatly in size among plant and animal viruses. While comoviruses have small peptide VPgs, comparable in size to those of the picornaviruses (Table 2), the VPgs of other plant viruses are larger proteins. These include the 188-amino-acid (aa) potato virus Y [86]; those of the luteoviruses are about 200 aa; and those of sobemoviruses are about 150 aa. The VPg of human norovirus, popularly known for causing nausea in cruise ship passengers, is about 132 aa and can be uridylylated at Tyr 27 [87].

The low sequence conservation seen in larger VPg proteins, as for those of caliciviruses, has complicated their analysis. The ones identified do not contain discernable characteristic peptides that would allow comparison with the small VPgs, although a positively charged area in the unstructured N-terminus of norovirus VPg mediates RNA binding [88]. It would be interesting to scan these proteins with the HMMs developed for the smaller VPgs [27].

The structural characterization of the larger VPgs is complicated by their inherent flexibility [89] and insolubility, a constant problem in dealing with proteins [86,90,91], which was not a problem when determining the NMR structures of chemically synthesized PV-VPg [30] and VPgpU [33]. All VPgs show structural flexibility, which is probably needed for nucleotidylation and priming.

In addition to their size, the caliciviruses differ from the picornaviruses in that they are covalently bound through a G residue at the 5′-end nucleotides of their RNAs [65]. Moreover, caliciviruses have VPgs bound to the 5′ ends of the full-length genomes, as well as a subgenomic RNA consisting only of structural genes VP1 and 2 [92]. Comoviruses also have VPgs at the 5′ ends of both their RNAs (only RNA 1 is shown in Figure 1).

The interaction of large VPgs in initiating translation from IRES elements is also different [93] from that in picornaviruses, where the removal of VPgs from transcribed RNA before translation enhances early (but not final) virus replication [37]. Structurally, the VPgs of caliciviruses have a compact helical core and some overall structural similarity within their group [94], but the sequences are diverse. A tyrosine for nucleotidylation has been identified in a structured loop in the VPg of porcine sapovirus [95], but RNA attachment to a reactive Y still needs to be clarified for most of these larger VPgs. Due to difficulties in cultivating the human norovirus, identifying the binding site to RNA or even the exact size of the bound protein proved difficult. The VPgs of cultivatable feline calicivirus and murine norovirus have been somewhat more amenable, but their NMR structures indicate that molecular details of their role in viral replication remain to be determined [94].

## 2. Conclusions

Although enteroviruses—omnipresent human pathogens—were the first viruses characterized as having a peptide linked to their RNA genomes, other genera of Picornaviridae have VPgs that are similar in size and PCPs. These VPgs replace the cap structures common to cellular RNAs and play a role in directing transcription and intracellular transport of their bound nucleic acids. The sequences and predicted structures of small VPgs from other viruses, which bear some similarity to those of picornaviruses, suggest roles for free lysine side chains in binding to RNA elements and interacting with viral and cellular proteins. Potyviruses and caliciviruses have large protein VPgs, which can have multiple roles in replication.

### Future Perspective

The viral proteins linked to the genome (VPg), including the longer ones of the human, animal and plant viruses, will be a source of research for many years to come. Their potential for antiviral drug design is only beginning to be explored.

## Figures and Tables

**Table 1:** Examples of VPg sequences from enteroviruses and those of other picornavirus genera, including multiple-copy VPgs from FMDV, mosavirus [25] and aquamavirus [26]. For colored iceLogos derived from multiple sequences, see [27]. The physicochemical property (PCP) consensus of enterovirus groups was calculated as described previously [24,28] using the five vectors derived from the multidimensional scaling of 237 physicochemical properties of the amino acids [29]. The sequence of EV-D68 (first called HRV 87 [22] from GenBank: WXG26576.1) shows more similarity to enteroviruses than to other rhinoviruses. Some rhinoviruses in turn show similarity to other genera of picornavirus sequences as they include negatively charged glutamic acid (E) residues. The sequences following VPg into the 3C protein area of the PV-1 and CVB3 are shown, as their similar PCPs suggest that there might have been 2 VPgs in the ancestral genes of the enteroviruses. The conserved 5-amino-acid site for uridylylation at the N-terminus is underlined, and the multiple lysine (K) residues throughout the rest of the peptide, which are a conserved feature of all VPgs, are highlighted.

Picornavirus	Sequence	IEP/Net Charge at pH 7
**Enterovirus VPgs:**		
**PV1-3**	** G ** ** A ** ** YTG ** **LP** **N** **KK** **P** **N** **VP** **T** **I** **R** **T** **A** **K** **V** **Q**	**10.9/4**
**HEV71**	**GAYSG**APKQVLKKPALRTATVQ	**10.9/4**
**CVA24**	**GAYTGLP**NKK**P**S**VPT**V**RTA**K**VQ**	**10.9/4**
**CVA21**	**GAYTGLP**NKK**P**S**VPTIR**V**A**K**VQ**	**10.9/4**
**CVB3**	**GAYTG**V**PN**QK**P**R**VPT**L**R**Q**A**K**VQ**	**11.5/4**
**CVB6**	**GAYTGMPN**QK**P**K**VPT**L**R**Q**A**K**VQ**	**10.9/4**
**EchovirusB**	**GAYTGMPN**QK**P**K**VPT**L**R**Q**A**K**VQ**	**10.9/4**
		
**PCP Consensus**	**GAYTG** **LPNQ** **K** **P** **K** **VPTIRTA** **K** **VQ**	**10.9/4**
		
**EV-D68 (HRV87)**	** GPYTG ** **IPNP** **K** **P** **K** **VPSLRTA** **K** **VQ**	**10.9/4**
		
**Rhino2**	**GPYSG**E.PKPKTKVPE.RRIVAQ	**10.4/3**
**Rhino14**	**GPYSG**NPPHNKLKAPTLRPVVVQ	**10.7/3**
**Rhino16**	**GPYSG**E.PKPKTKVPE.RRVVAQ	**10.4/3**
**Rhino89**	**GPYSG**E.PKPKSRAPE.RRVVTQ	**10.7/3**
		
**PV1 following**	**GP**GFD**Y**AVAMAKRNIVTATTSKG	**10.2/2**
**CVB3 following**	**GP**AFEFAVAMMKRNSSTVKTEYG	**9.6/1**
		
**Kobuvirus**		
**Aichi virus**	** AAYSA ** ISHQ K P K P K SQKPVPTRHIQRQ	**11.6/6.7**
**Sicinivirus (chick)**	** AAYTG ** ------ K PPTR K Q K RDPEPQ	**10.1/3**
		
**Aphthovirus 3 VPgs:**		
**FMDV-VPg1**	**GPYAG**PLERQRPLKVRAKLPR**Q**E	**11.3/4**
**FMDV-VPg2**	**GPYAG**PMERQKPLKVKARAPVVKE	**10.6/4**
**FMDV-VPg3**	**GPYAG**PVKKPVALKVKAKNLIVTE	**10.5/4**
		
**Mosavirus 2 VPgs:**		
**European Roller**	**GPYCG**ACKRKAPVLKKTVAE	**10/4**
**Picornavirus**	**GPYSG**MPRATPKKLKKVVVQ	**11/5**
		
**Aquamavirus 2 VPgs:**		
**Ringed seal**	**SAYEG**CSTRKTARQLARSVVGE	**9.7/2**
**Picornavirus**	**GAYDG**NVKRTTARELARKAIPSEQ	**10.2/2**

**Table 2:** Examples of VPgs of Comoviridae (plant pathogens) and dicistroviruses isolated from insects. The bold S at the N termini of the Comoviridiae sequence signifies the probable location of the RNA binding site, which is not generally known for the dicistroviruses. The number of repeats in the dicistroviuses is indicated in () in the latter. For a more complete list of VPg sequences from dicistroviruses and iceLogos illustrating conservation, see [27,61]. There are slight sequence differences between VPg copies in the same discistrovirus, as illustrated for CrPV and PSIV; residues **S4**, **T9** were shown by mass spectroscopy to be uridylylated in the first CrPV VPg shown [15].

Virus	VPg Sequence	IEP/Net Charge at pH7 Comoviridae (Plant)
**Comoviridae (plant)**		
Cowpea mosaic virus	**S**RKPNRFDMQQYRYNNVPLKRRVWADAQMSLDQ	10.8/4 *
Squash mosaic virus	**S** R K PNRFDVAQYRYRNVPL K RRQWADAQMSLDH	11.3/5
Red clover mottle	**S** R K PNRFEVQQYRY K NVPLTRRSWGNAQMSLDQ	11.3/5
Bean pod mottle virus	**S** R K PNRYEVSQYRYRNVPI K RRAWVEGQMSFDQ	10.9/5
Broad bean true mosaic	**S** R K PNRHDQEQHRYRNVPLTRRNWATAQMSLHQ	12.2/5.3
Pepper mild mosaic **	**S** KK PNRYDVSSY K YRNVPLRQRAWAQAQMSIDQ	10.7/5
**Dicistroviridae (insect)**		
Plautia stali	SQEKEGVISRCKIE (3×)	6.5/0
intestinal virus (PSIV)	SQEKIGSVSRVRVE	10/1
	SQEKLGAIPAVKIE	7/0
Himetobi P virus (HIPV)	SYDVGNIKPTRTE (4×)	7.0/0
Cricket paralysis (CrPV)	VGS**S**GDNK**T**QKISKRVVE (4×)	10.3/2
	VGG**S**GDVK**T**TKPAKTAVE	9.7/1
	VGS**S**GDSK**T**MKNKITKVE	10.2/2
	VGS**S**GDSK**T**QKQRNTKVE	10.3/2
Solenopsis invicta (SINV-1)	AAT**S**GDCM**T**KVKPRVILE (5×)	8.9/1

* sequence demonstrated [62]. Other Comoviridae sequences are from gene sequencing by homology; ** sequence is identical to that of Andean potato mottle virus.
